# Tumor suppressors LKB1 and SMARCA4 functionally interact to regulate gene expression across diverse biological processes in lung cancer

**DOI:** 10.3389/fcell.2026.1685342

**Published:** 2026-03-17

**Authors:** Mohammed Bourouh, Jinhong Kim, Paola A. Marignani

**Affiliations:** 1 Department of Biochemistry and Molecular Biology, Faculty of Medicine, Dalhousie University, Halifax, NS, Canada; 2 Department of Biological Sciences, Faculty of Science, Thompson Rivers University, Kamloops, BC, Canada

**Keywords:** gene expression, liver kinase B1, lung cancer, metabolism, single-cell RNA-seq, SWI/SNF-related matrix associated actin-dependent regulator of chromatin subfamily A, LKB1, SMARCA4

## Abstract

**Introduction:**

The tumor suppressor kinase liver kinase B1 (LKB1) is known to regulate the activity of the metabolic sensor AMP-activated protein kinase (AMPK), which, under energy stress, shifts metabolism from anabolism to catabolism, thus linking LKB1 to AMPK-mediated gene expression. Coupled with its role as a tumor suppressor kinase, LKB1 is an important metabolic regulator implicated in multiple malignancies and is frequently mutated in lung cancer. Previously, we discovered that LKB1 binds to the switch/sucrose non-fermenting (SWI/SNF) chromatin remodeling ATP-dependent helicase subunit SWI/SNF-related, matrix-associated, actin-dependent regulator of chromatin, subfamily A, member 4 (SMARCA4), directly linking LKB1 to gene expression. How LKB1 and SMARCA4 collaborate to regulate gene expression in lung cancer has not been well characterized.

**Methods:**

We used an *in silico* approach to explore how LKB1 and SMARCA4 may cooperate to regulate gene expression. We analyzed our previous single-cell RNA-seq (scRNA-seq) dataset from four lung cancer cell lines with differential *LKB1* and *SMARCA4* expression status to identify genes regulated by both LKB1 and SMARCA4. We correlated our results using bulk RNA-seq results from human lung tumors.

**Results:**

We show that LKB1 and SMARCA4 likely function together to regulate gene expression in multiple biological processes in lung cancer cell lines. Gene expression profiles from LKB1- and SMARCA4-mutant cells are similar, suggesting that LKB1 and SMARCA4 function in a linear pathway to regulate gene expression. Furthermore, we observed similar results in human lung tumors, particularly in late-stage disease.

**Discussion:**

We propose a model where LKB1 acts as a nexus between metabolism and gene expression, acting via the SMARCA4–SWI/ SNF complex to regulate gene expression in lung cancer.

## Introduction

Metabolic reprogramming plays a crucial role in cancer cells’ ability to thrive in nutrient-poor environments and regulate gene expression through metabolic signaling intermediates. Epigenetic enzymes utilize key metabolic intermediates such as ATP, acetyl-CoA, and S-adenosylmethionine (SAM) to modulate gene expression through histone modifications (Gut and Verdin, 2013; Kottakis et al., 2016; Sutendra et al., 2014). Epigenetic histone modifications alter the chromatin landscape, generating landmarks at gene regulatory elements to differentiate transcriptionally active chromatin regions from transcriptionally silent regions. The switch/sucrose non-fermenting (SWI/SNF) chromatin remodeling complexes, for example, hydrolyze ATP to displace nucleosomes, mobilize histones, and open chromatin for regulating gene expression (Bultman et al., 2000; Kwon et al., 1994). The availability of ATP, acetyl-CoA, and SAM can drastically alter the global chromatin landscape, and the availability of these cofactors is determined by the cells’ metabolic state. This mechanism enables cells to leverage metabolic output to influence cellular responses via epigenetic regulation of gene expression. Mutations in tumor suppressor genes that disrupt metabolic pathways can result in aberrant epigenetic changes commonly associated with malignant transformation.

Important regulators of metabolism and epigenetics are tumor suppressors liver kinase B1 (LKB1) and SWI/SNF-related, matrix-associated, actin-dependent regulator of chromatin, subfamily A, member 4 (SMARCA4), which are often mutated or lost in non-small-cell lung cancer (NSCLC) (Rodriguez-Nieto and Sanchez-Cespedes, 2009; Marignani, 2005). Interestingly, *LKB1* and *SMARCA4* are also located in close proximity on chromosome 19p13.3, and loss of heterozygosity (LOH) is frequently observed (Rodriguez-Nieto and Sanchez-Cespedes, 2009; Marignani, 2005). The best characterized role of LKB1 is in energy metabolism, where LKB1 activates AMP-activated protein kinase (AMPK) to shift metabolic pathways from anabolism to catabolism (Bourouh and Marignani, 2022; Hawley et al., 2003; Shaw et al., 2004). SMARCA4 is the ATP-dependent helicase subunit of the SWI/SNF chromatin remodeling complex, hydrolyzing ATP to displace nucleosomes, opening chromatin for transcription factors to bind gene regulatory elements (Bultman et al., 2000; Kwon et al., 1994). Both *SMARCA4*- and *LKB1*-mutant NSCLC cases are associated with smokers and, consequently, with *KRAS* mutations. *LKB1* is mutated in approximately 20% of NSCLC cases (La Fleur et al., 2019; Gao et al., 2022), while *SMARCA4* is mutated in 8% of NSCLC cases, with 39% of those cases representing *LKB1–SMARCA4* co-mutation (Medina et al., 2008; Schoenfeld et al., 2020).

Our laboratory previously discovered that nuclear localized LKB1 binds to SMARCA4 *in vivo*, specifically to its helicase domain, promoting SMARCA4 ATPase activity, independent of LKB1’s catalytic function (Marignani et al., 2001). ATPase experiments *in vitro* showed that in the presence of DNA, the ATPase activity of SMARCA4 is three-fold higher in the presence of LKB1 compared with the presence of SL26, a catalytically deficient mutant of LKB1 first identified in Peutz–Jeghers syndrome (Hemminki et al., 1998). This strongly suggests that the binding of SMARCA4 to LKB1 is necessary for the ATPase activity of SMARCA4 and that the ATP-dependent chromatin remodeling function of SMARCA4 is reliant on LKB1 (Marignani et al., 2001). We have also shown that the tumor suppressor function of SMARCA4 is partially dependent on LKB1. When *SMARCA4* is expressed in SW13 cells, a *SMARCA4*-mutant cell line that expresses endogenous *LKB1*, SW13 cells undergo cell cycle arrest. The cell cycle arrest is suppressed when *SMARCA4* is co-expressed with *SL26* but not *LKB1*, indicating that the catalytic activity of LKB1 is required for SMARCA4-mediated cell cycle arrest (Marignani et al., 2001). In a later study, we discovered that catalytically deficient *LKB1* mutants promote the expression of *CYCD1* by directly binding to the *CYCD1* promoter (Scott et al., 2007). Since SMARCA4 promotes the expression of *CDKN1A* (*P21*), leading to the inhibition of CDK4–CYCD1, which reduces the phosphorylation of retinoblastoma (RB), thus repressing E2F transcription factors and ultimately inhibiting the expression of G1/S factors (Kang et al., 2004). More recently, the loss of binding between LKB1 and SMARCA4 has been shown to result in PRC2-dependent transcriptional inhibition through increased H3K27me3. This, in turn, leads to upregulation of oxidative stress pathways and dysregulation of amino acid metabolism (Mével-Aliset et al., 2025).

Indirect observations implicate LKB1 and SMARCA4 in lipid metabolism, a well-characterized function of LKB1. The LKB1–AMPK pathway can regulate the expression of genes involved in fatty acid (FA) biosynthesis. Phosphorylation of sterol regulatory element-binding protein 1 (SREBP1), a transcriptional co-activator, by AMPK results in inhibition of SREBP1, leading to deregulation of FA biosynthesis genes (Seo et al., 2015; Li et al., 2011). SMARCA4 can bind to SREBP1, suggesting that SMARCA4 plays a role in regulating the expression of FA biosynthesis genes, indirectly linking SMARCA4 to LKB1 functions (Li et al., 2018a). In addition to SREBP1, the LKB1–AMPK signaling pathway is involved in regulating β-oxidation through peroxisome proliferator-activated receptor (PPAR) nuclear receptors. Here, LKB1–AMPK signaling activates PPARα in skeletal muscle cells, which leads to increased transcription of genes involved in β-oxidation (Juszczak et al., 2020; Lee et al., 2006). SWI/SNF complexes also play a role in the PPAR pathway as the SWI/SNF complex is recruited to *PPARγ* target genes (Salma et al., 2004). These results indirectly suggest that the interaction between LKB1 and SMARCA4 regulates lipid metabolism.

Finally, LKB1 and SMARCA4 both regulate transcription mediated by the ERα receptor (Nath-Sain and Marignani, 2009; DiRenzo et al., 2000). LKB1 binds to ERα independently of catalytic activity, although LKB1 catalytic activity is required for transcription of ERα target genes. Interestingly, ERα can recruit SMARCA4 to ERα target genes, where this recruitment is dependent on histone acetylation. Here, LKB1 and SMARCA4 may function together to regulate the expression of ERα target genes, with histone modifications playing an important role (Nath-Sain and Marignani, 2009; DiRenzo et al., 2000).

LKB1 has also been linked to chromatin organization independently of SMARCA4 (Kottakis et al., 2016; Pierce et al., 2021). One study examined the effect of Lkb1 on chromatin accessibility using the assay for transposase-accessible chromatin using sequencing (ATAC-seq) after restoring *Lkb1* expression in *Lkb1*-mutant mouse lung tumor cells and found that more than 30,000 genomic loci exhibited changes in chromatin accessibility (Pierce et al., 2021). Furthermore, another study linked *Lkb1* loss in mouse lung tumors to upregulation of SAM production, leading to global histone methylation and repression of retrotransposons (Kottakis et al., 2016).

The consequences of the *Lkb1* chromatin-remodeling function have been observed in collaboration with *Kras*-mutant lung cancer in mouse models. It was observed that *Lkb1* loss in *Kras* lung cancer mouse models exhibits loss of H3K27me3 and gain of H3K27ac and H3K4me3 histone modifications. These modifications correlated with the transition from the lung adenocarcinoma subtype to squamous cell carcinoma, indicating that this epigenetic change was facilitated through *Lkb1* loss and suggesting that *Lkb1* loss impacted differential subtypes of lung cancer progression mediated by histone modifications (Zhang et al., 2017).

Independent of *Kras*, *Lkb1* functions to regulate chromatin dynamics in pancreatic β cells. *Lkb1* loss was correlated with the dysregulation of *Foxa, Mafa*, and *Rfx6* transcriptional regulatory elements. This highlights a specific role of *Lkb1* in regulating chromatin dynamics across a variety of cells, independent of *Kras* and *Smarca4* (Haberman et al., 2024).

These studies highlight the functional role of LKB1 in regulating chromatin remodeling via SMARCA4; however, the involvement of SMARCA4 in LKB1-dependent pathways and gene expression regulation has not been well characterized. To elucidate the functional relationship between LKB1 and SMARCA4 in lung cancer, we conducted single-cell RNA-seq (scRNA-seq) on four lung cancer cell lines with varying *LKB1* and *SMARCA4* mutation statuses (Carretero et al., 2004; Kim et al., 2021). Our analysis revealed that LKB1 and SMARCA4 function together in a linear pathway to modulate overlapping gene expression profiles in lung cancer cells. Notably, we observed that the expression profiles associated with *LKB1* and *SMARCA4* become apparent in late-stage human lung tumors. Our findings support a model wherein LKB1 functions dually, both as a metabolic regulator, acting through AMPK signaling, and as a transcriptional regulator, acting through the SMARCA4–SWI/SNF chromatin remodeling complex. Consequently, our model positions LKB1 as a crucial nexus bridging metabolic processes and gene expression regulation in lung cancer.

## Results

### LKB1 and SMARCA4 regulate expression of overlapping genes

**FIGURE 1:**
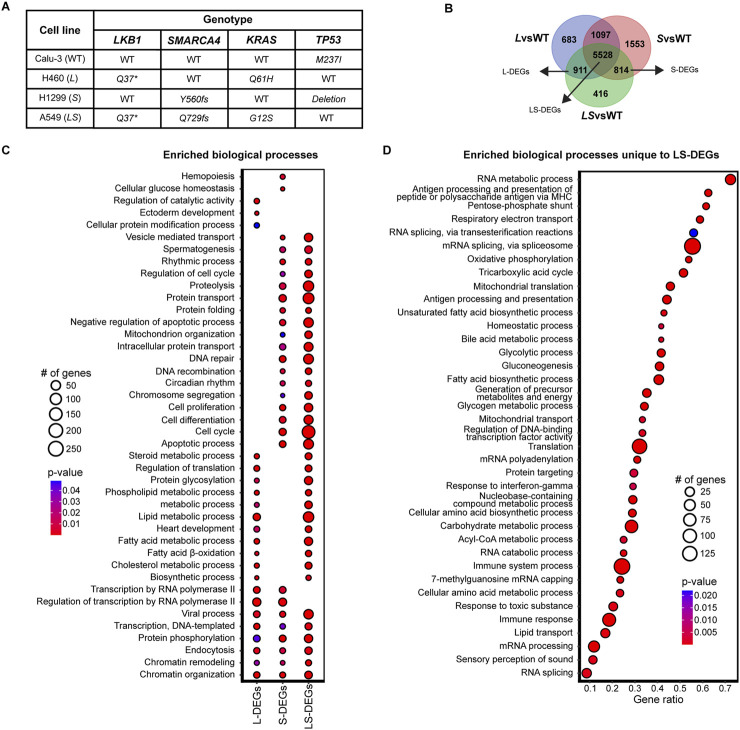
scRNA-seq on lung cancer cell lines identifies LKB1 and SMARCA4 gene expression signatures. **(A)** Genotype of lung cancer cell lines (Calu-3, H460, H1299, and A549) used for scRNA-seq. **(B)** Venn diagram representing the classification of DEGs: L-DEGs, S-DEGs, and LS-DEGs by comparing *L*vsWT, *S*vsWT, and *LS*vsWT DEGs. Number of DEGs classified as L-DEGs (n = 911), S-DEGs (n = 814), and LS-DEGs (n = 5,528). **(C,D)** Dot plot of enriched biological process (BP) terms from GSEA. Enriched BP terms for L-DEGs, S-DEGs, and LS-DEGs **(C)** and unique to LS-DEGs **(D)**. Gene ratio in D calculated by the number of genes/term size. Dot size represents the number of genes identified in each BP term; color represents the p-value, with red representing a low p-value and blue representing a high p-value. Only BP terms that are statistically significant (p < 0.05) and have >3 genes are shown.

To characterize the interaction between LKB1 and SMARCA4, we analyzed our scRNA-seq dataset from four well-characterized lung cancer cell lines with varying *LKB1* and *SMARCA4* expression statuses: Calu-3, H460, H1299, and A549 (Figure 1A) (Carretero et al., 2004; Kim et al., 2021). Calu-3 cells (WT) are wild-type for both *LKB1* and *SMARCA4*; therefore, they are referred to as WT. H1299 cells (*S*) are *SMARCA4*-deficient, H460 cells (*L*) are *LKB1*-deficient, and A549 cells (*LS*) are *LKB1-* and *SMARCA4*-deficient (Figure 1A) (Blanco et al., 2009). None of these mutations produce functional protein (Carretero et al., 2004; Blanco et al., 2009; Medina et al., 2005).

To classify differentially expressed genes (DEGs), we compared log_2_ fold change (log_2_FC) of all 11,002 DEGs detected in our scRNA-seq dataset between *L*vsWT, *S*vsWT, and *LS*vsWT (Supplementary Table S1). We classified DEGs as LKB1-specific (L-DEGs), SMARCA4-specific (S-DEGs), or LKB1-SMARCA4-specific (LS-DEGs) based on the DEG expression profile. We reasoned that L-DEGs are detected only when *LKB1* is mutated (*L*vsWT and *LS*vsWT, n = 911) and S-DEGs are detected only when *SMARCA4* is mutated (*S*vsWT and *LS*vsWT, n = 814) (Figure 1B). LS-DEGs comprise the largest classification (n = 5,528, ∼50% of genes detected) and represent DEGs detected when either *LKB1* or *SMARCA4* is mutated (*L*vsWT, *S*vsWT, and *LS*vsWT) (Figure 1B). Therefore, our results suggest that LKB1 and SMARCA4 are important regulators of gene expression and may largely function together to regulate the expression of common genes.

### LKB1 and SMARCA4 have considerable overlapping functions

We then performed gene set enrichment analysis (GSEA) on L-DEGs, S-DEGs, and LS-DEGs to identify overrepresented Gene Ontology (GO) terms within the biological process (BP) category (see Materials and methods) (Supplementary Table S2). L-DEGs were enriched in biological processes related to metabolic pathways, particularly in fatty acid and cholesterol metabolic processes, consistent with well-characterized LKB1 functions (Figure 1C) (Bourouh and Marignani, 2022; Scott et al., 2007; Hendricks et al., 2004; Gupta et al., 2020). S-DEGs were enriched in diverse biological processes, including protein transport, cell cycle, cell proliferation, and DNA repair (Figure 1C). L-DEGs and S-DEGs displayed overlapping enrichment in biological processes related to gene expression, depicted by overlapping functions in transcription, chromatin organization, and chromatin remodeling (Figure 1C).

LS-DEGs represented the overwhelming majority of classified DEGs. If these DEGs represented overlapping functions of LKB1 and SMARCA4, LS-DEGs would be enriched for similar BP terms as L-DEGs and S-DEGs. Enriched BP terms for LS-DEGs showed significant overlap with enriched biological processes of L-DEGs and S-DEGs, suggesting that LS-DEGs were enriched for genes that represent LKB1 and SMARCA4 functions (Figure 1C). In addition, LS-DEGs also exhibited unique enrichment in various biological processes implicated by LKB1 and SMARCA4 (Figure 1D). Many overrepresented BP terms corresponded to metabolic processes, but we also observed enrichment in translation, transcription, immunity, and cell cycle biological processes (Supplementary Figure S1A). Therefore, our workflow identified both unique and overlapping functions of LKB1 and SMARCA4, consistent with their previously characterized roles (Bourouh and Marignani, 2022; Scott et al., 2007; Hendricks et al., 2004).

### *LKB1-* and *SMARCA4*-mutant human tumors exhibit similar expression profiles

**FIGURE 2:**
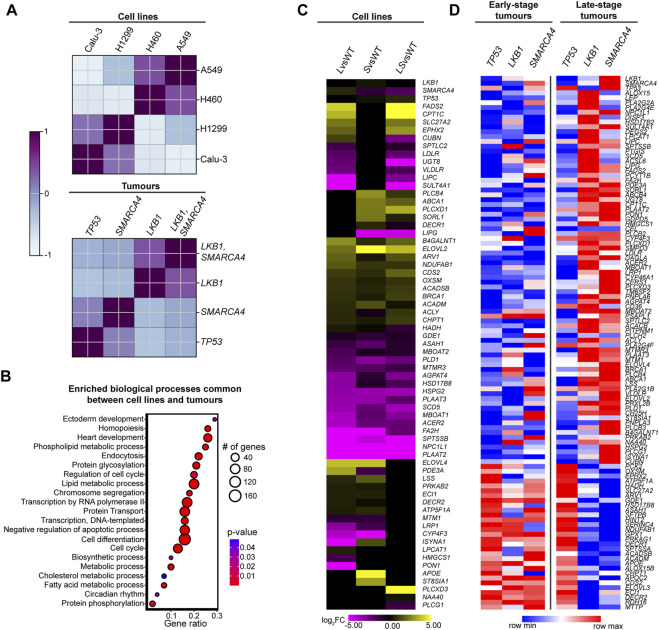
*LKB1*- and *SMARCA4*-mutant lung tumors exhibit similar expression profiles. **(A)** Correlation analysis between scRNA-seq of lung cancer cell lines Calu-3, H460, H1299, and A549 and *TP53*, *LKB1*, *SMARCA4*, and *LKB1/SMARCA4* human lung tumors from the cBioPortal database. Cell line or genotype of tumor is listed on the top and right of two pair-wise matrix plots. Color represents z-score of the pair-wise matrix plots. Blue represents low z-scores (low correlation), while purple represents high z-scores (high correlation). **(B)** Dot plots of enriched BP terms common between cell lines and tumors (L-DEGs, S-DEGs, LS-DEGs, and *LKB1-* and *SMARCA4*-mutant tumors). The gene ratio represents the number of genes/term size. Dot size represents the number of genes identified in the term, color represents the *p*-value, with red representing a low *p*-value and blue representing a high *p*-value. Only BP terms that are statistically significant (*p* < 0.05) and have >3 genes are shown. **(C,D)** Heatmap plot of expression of DEGs identified from GSEA from *LKB1* and *SMARCA4* early-stage and late-stage tumors involved in lipid metabolic process. **(C)** The heatmap plot represents expression of genes from *L*vsWT, *S*vsWT, and *LS*vsWT using scRNA-seq data (purple/yellow). Yellow represents high expression, and purple represents low expression with scale of −5 to 5 log_2_FC. DEGs not detected or have no change in expression are represented in black. **(D)** The heatmap plot represents expression of genes from *TP53*, *LKB1*, and *SMARCA4* early-stage and late-stage tumors (red/blue). Red represents high expression, and blue represents low expression with scale of row min–row max.

The largely overlapping BP terms suggest that LKB1 and SMARCA4 function together to regulate gene expression of common pathways in cultured cells. To explore the translational consequences of this interaction in lung cancer, we asked whether our scRNA-seq results could be recapitulated using lung tumor transcriptional data from the cBioPortal cancer genomics database (Cerami et al., 2012; de Bruijn et al., 2023; Gao et al., 2013). *KRAS-LKB1*-mutant lung tumors exhibit distinct transcriptional and phenotypic properties compared with *KRAS-TP53*-mutant lung tumors (Skoulidis et al., 2015); however, the relationship between *LKB1-* and* SMARCA4*-mutant tumors has not been investigated. We examined bulk RNA-seq data of tumors from the cBioPortal database, selecting tumors with genetic backgrounds similar to the cell lines used in our scRNA-seq analysis: Calu-3, H460, H1299, and A549, with *TP53*-mutant tumors representing Calu-3 (Figure 1A; Supplementary Table S3). We first explored the overall transcriptional similarity between cell lines and primary lung tumors. We performed a correlation analysis to examine transcriptomic relationships among the four tumor types, namely, *TP53*, *LKB1*, *SMARCA4*, and *LKB1/SMARCA4* double mutants, and compared the correlation matrices of these tumors with those of the cell lines used in our scRNA-seq data. We observed transcriptional similarity between cell lines and tumors mutant for *LKB1* (H460 and A549) and between *LKB1*- and *LKB1-SMARCA4*-mutant tumors (Figure 2A). Moreover, *SMARCA4*- and *TP53*-mutant tumors expression profiles are correlated, such as the comparison between H1299 (*SMARCA4*-mutant) and Calu-3 cells (*TP53*-mutant) (Figure 2A). These results revealed transcriptional similarity between the cell lines used in our scRNA-seq and primary lung tumor bulk RNA-seq data from cBioPortal.

To investigate pathways implicated in *LKB1*- and *SMARCA4*-mutant tumors, we performed GSEA on DEGs from *LKB1*- and *SMARCA4*-mutant tumors, using the same pipeline as the GSEA used for scRNA-seq data (Supplementary Table S4). We then compared enriched biological processes between cell lines and tumors. Metabolic and cell cycle BP terms are common between *LKB1-* and *SMARCA4*-mutant lung tumors and cell line GSEA results, with genes that function in the lipid metabolic process also enriched (Figure 2B; Supplementary Figure S2A). We next compared the expression of enriched DEGs that comprise the lipid metabolic process identified in GSEA of tumors between our scRNA-seq and cBioPortal bulk RNA-seq datasets using a heatmap plot (Figures 2C,D; Supplementary Table S5). Most of the genes exhibit expression patterns characteristic of LS-DEGs (Figure 2C) and display similar expression levels in *L*vsWT, *S*vsWT, and *LS*vsWT comparisons. There is individual contribution from both LKB1 and SMARCA4 to the expression of genes linked to lipid metabolism, but the majority of DEGs are impacted by both *LKB1* and *SMARCA4* loss. We also applied our workflow to investigate the LKB1–SMARCA4 transcriptional phenotype, using WT tissue and *KRAS*-mutant tumors as controls. When comparing *LKB1*- and *SMARCA4*-mutant tumors to WT tissue, *TP53-*, and *KRAS*-mutant tumors, we observed an inverse relationship with respect to correlation scores of enriched GO terms in *LKB1-* and *SMARCA4*-mutant tumors compared to WT, *KRAS-*, and *TP53*-mutant tumors (Supplementary Table S6). Furthermore, enriched GO terms in *LKB1-* and *SMARCA4*-mutant tumors exhibit similar enrichment scores in all three comparisons (Supplementary Table S6). Therefore, these results suggest that there is some transcriptional similarity between *LKB1-* and *SMARCA4*-mutant tumors (Supplementary Table S6).

We next examined the expression profile of lipid metabolic process DEGs in *TP53*, *LKB1*, and *SMARCA4* early-stage and late-stage lung tumors (Figure 2D). Early-stage *TP53-*, *LKB1-* and *SMARCA4*-mutant tumors predominantly exhibit downregulation of lipid metabolic process genes (Figure 2D). Interestingly, in late-stage tumors, we observed that *LKB1*- and *SMARCA4*-mutant tumors demonstrate strikingly similar expression profiles of genes involved in lipid metabolic processes, particularly upregulation of several genes (Figure 2D). Furthermore, this expression signature differs from that of late-stage *TP53*-mutant tumors and early-stage *LKB1-* and *SMARCA4-*mutant tumors, suggesting that *LKB1-* and *SMARCA4*-mutant tumors exhibit similar phenotypes that emerge only during late-stage disease progression (Figure 2D). To determine whether other pathways exhibit a similar expression signature, we examined DEGs that comprised the cell cycle BP term, and we observed a similar gene expression pattern between *LKB1-* and *SMARCA4*-mutant tumors, with cell cycle genes largely upregulated in late-stage tumors and in cell lines (Supplementary Figure S2B,C). These results indicate that LKB1 and SMARCA4 function cooperatively to regulate tumorigenesis in lung cancer.

### SMARCA4 and LKB1 regulate gene expression in a linear pathway

**FIGURE 3:**
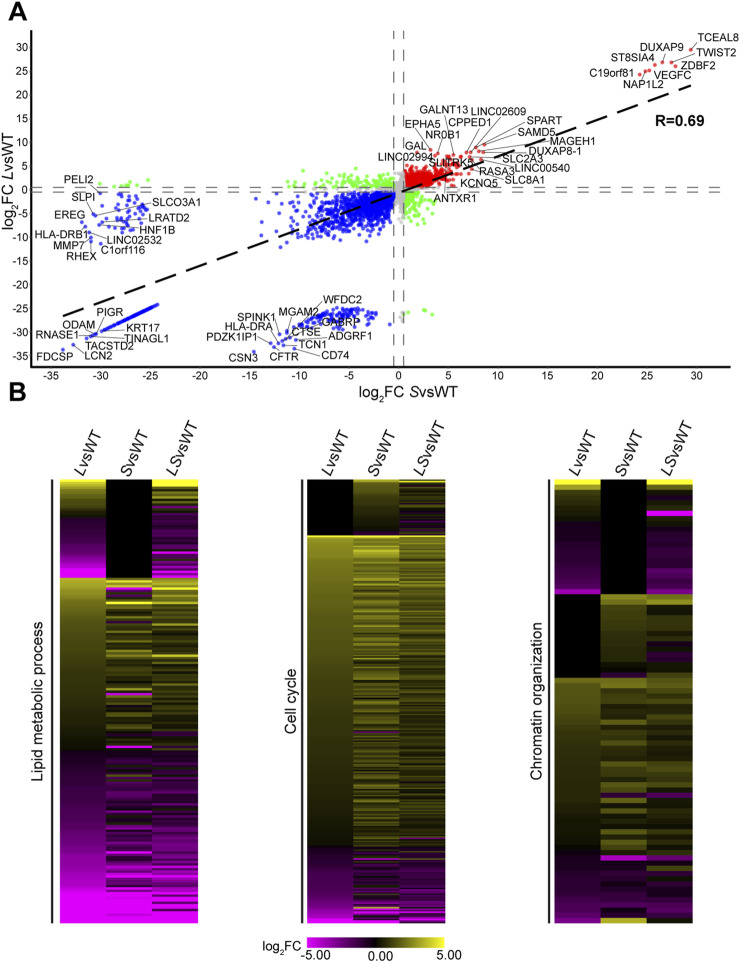
LKB1 and SMARCA4 regulate a similar expression program. **(A)** Scatterplot of DEG expression in *L*vsWT compared with that in *S*vsWT. Red represents upregulated genes above the log_2_FC = 0.5 threshold, and blue represents downregulated genes below the log_2_FC = −0.5 threshold, while gray represents genes within log_2_FC 0.5 and −0.5 thresholds. Green dots represent inversely related genes. Black dashed lines represent correlation trend lines (R = 0.69), and dashed gray lines represent log_2_FC = 0.5 and −0.5 thresholds. Top and bottom 15 genes beyond the thresholds from *L*vsWT and *S*vsWT are labeled. **(B)** Heatmap plot displaying expression of DEGs identified in lipid metabolic process, cell cycle, and chromatin organization from GSEA of L-DEGs, S-DEGs, and LS-DEGs in *L*vsWT, *S*vsWT, and *LS*vsWT. Yellow represents high expression, and purple represents low expression with scale of −5 to 5 log_2_FC. DEGs not detected or have no change in expression are represented in black.

The transcriptional similarity between our scRNA-seq data from cultured cells and bulk RNA-seq data from human lung tumors establishes a link between LKB1 and SMARCA4 in transcriptional regulation. We noticed that the majority of DEGs from scRNA-seq were classified as LS-DEGs, encompassing ∼50% of all genes detected in our scRNA analysis, indicating that LKB1 similarly plays an important role in regulating gene expression as SMARCA4 (Figure 1B). Furthermore, L-DEGs outnumbered S-DEGs, suggesting a greater requirement for LKB1 in regulating gene expression. Since LKB1 has been shown to bind and promote the ATPase activity of SMARCA4 and LKB1 localizes to both the nucleus and cytoplasm (Marignani et al., 2001; Nezu et al., 1999; Boudeau et al., 2003), we postulated that the transcriptional regulatory function of LKB1 could be mediated primarily through SMARCA4. To evaluate this, we compared the expression of LS-DEGs present in *L*vsWT with that in *S*vsWT using a scatter plot (Figure 3A). We observed that LS-DEGs were similarly expressed in *L*vsWT compared with *S*vsWT, exhibiting a strong correlation of expression (R = 0.69) (Figure 3A). Interestingly, our analysis revealed that only 454 genes out of 5,528 (8.2% of genes) were inversely regulated, suggesting that LKB1 and SMARCA4 primarily co-regulate LS-DEGs in a linear pathway (Supplementary Table S1).

To evaluate the consequences that *LKB1* and *SMARCA4* loss have on gene expression, we visualized the expression pattern of DEGs identified in our GSEA for lipid metabolic process, cell cycle, and chromatin organization in *L*vsWT, *S*vsWT, and *LS*vsWT using heatmap plots (Figure 3B; Supplementary Table S7). We observed that L-DEGs involved in the lipid metabolic process are generally downregulated and exhibit similar expression in *L*vsWT compared with *LS*vsWT. LS-DEGs that function in the lipid metabolic process displayed comparable differential expression when either *LKB1* or *SMARCA4* was mutated, with an equivalent number of genes up- and downregulated, suggesting that loss of either *LKB1* or *SMARCA4* produced similar transcriptional consequences on genes implicated in the lipid metabolic process (Figure 3B). Likewise, *LKB1* or *SMARCA4* mutations had similar effects on the expression of genes associated with cell cycle and chromatin organization (Figure 3B). Loss of either *LKB1* or *SMARCA4* caused upregulation of cell cycle genes, and the magnitude of upregulation was similar between *LKB1* loss and *SMARCA4* loss. We also noticed a similar profile with genes that function in chromatin organization (Figure 3B). These results highlight the phenotypic similarity resulting from the loss of *SMARCA4* or *LKB1* on gene expression across diverse biological processes and suggest that LKB1 and SMARCA4 function within a linear pathway.

The similar transcriptional profile observed in multiple biological processes between *LKB1* and *SMARCA4* loss suggested that LKB1 and SMARCA4 largely function together to regulate gene expression (Mével-Aliset et al., 2025). To validate our results, we applied an unbiased approach by examining the expression of genes within our scRNA-seq dataset that are annotated to lipid metabolic process, cell cycle, and chromatin organization in the Gene Ontology Resource (Ashburner et al., 2000; Aleksander et al., 2023). We reasoned that if LKB1 and SMARCA4 function in a linear pathway with respect to gene expression regulation, most annotated genes from these biological processes would be classified as LS-DEGs and display similar expression profiles in *L*vsWT, *S*vsWT, and *LS*vsWT, consistent with the DEGs identified in our GSEA (Figure 3B). We visualized the expression profiles of genes annotated to lipid metabolic process, cell cycle, and chromatin organization BP terms and observed that loss of *LKB1* or *SMARCA4* results in a similar transcriptional phenotype (Supplementary Figure S3A; Supplementary Table S7). The majority of DEGs classified as LS-DEGs, while LKB1 and SMARCA4 provided distinct contributions to gene expression in these biological processes (Supplementary Figures S3A,B). In the lipid metabolic process, L-DEGs outnumber S-DEGs (n = 78 vs. n = 45), suggesting a greater requirement for LKB1 in lipid metabolism. Similarly, there is a greater contribution from SMARCA4 in chromatin remodeling, with S-DEGs outnumbering L-DEGs (n = 55 vs. n = 40).

To further delve into the overlapping function of LKB1 and SMARCA4, we also examined the expression of genes annotated to the immune response BP term (Supplementary Figures S1B,C). LKB1 has been implicated in immune response as human *LKB1*-mutant tumors are characterized by low immunogenicity, and *LKB1* loss is associated with high inflammatory characteristics (Koyama et al., 2016; Skoulidis et al., 2018). Tumors deficient in *LKB1* display gene expression profiles indicative of a suppressive immune tumor microenvironment, with reduced infiltration of CD8+/CD4+ T cells (Gao et al., 2022). This phenotype can be recapitulated with loss of AMPK activity, linking *LKB1* loss with diminished AMPK activity. This, coupled with the observation that LS-DEGs show strong enrichment for genes involved in immunity, suggests that SMARCA4 and LKB1 collaborate to regulate the expression of immune-related genes (Figure 1D; Supplementary Figures S1B,C). We found that L-DEGs enriched in immune function are downregulated (Supplementary Figure S1B), while S-DEGs and LS-DEGs show a similar number of up- and downregulated genes. Expression of most immune response genes is dependent on both *LKB1* and *SMARCA4* (n = 548 out of n = 985 DEGs, ∼55.6%), indicating that LKB1 and SMARCA4 may collaborate to regulate expression of immune response genes.

### SMARCA4 regulates the expression of LKB1-associated genes

**FIGURE 4:**
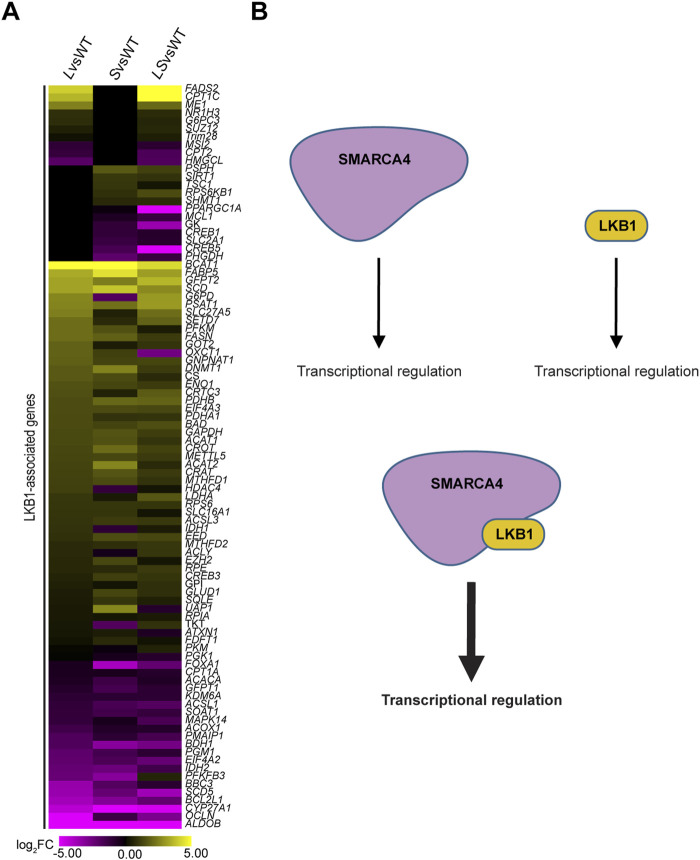
SMARCA4 regulates expression of LKB1-associated genes. **(A)** Heatmap plot showing the expression profile of LKB1-associated genes in *L*vsWT, *S*vsWT, and *LS*vsWT. Yellow represents high expression, and purple represents low expression with scale of −5 to 5 log_2_FC. DEGs not detected or have no change in expression are represented in black. **(B)** SMARCA4 and LKB1 have independent and cooperative roles in transcriptional regulation.

Our results indicate that LKB1 and SMARCA4 regulate the expression of genes involved in LKB1 pathways. If this is true, we predict that SMARCA4 would also regulate the expression of genes known to be associated with or be substrates of LKB1. Hence, we curated a list of LKB1-associated genes, particularly metabolic regulators, chromatin remodeling enzymes, epigenetic modifying enzymes, or genes differentially expressed in the *LKB1*-mutant context in multiple systems (mouse and human cell lines and tumors) (Kottakis et al., 2016; Pierce et al., 2021; Zhang et al., 2017; Timilshina et al., 2019). We examined the expression of LKB1-associated genes in *L*vsWT, *S*vsWT, and *LS*vsWT, and interestingly, we observed that the majority of DEGs are classified as LS-DEGs, indicating that SMARCA4 plays an important role in regulating transcription of LKB1-associated genes (Figure 4A; Supplementary Table S9). Furthermore, the expression of LKB1-associated genes was remarkably similar between *L*vsWT, *S*vsWT and *LS*vsWT, suggesting that LKB1 and SMARCA4 work together to regulate the expression of genes particularly implicated by LKB1 (Supplementary Table S9). These results suggest that LKB1 and SMARCA4 may function in a linear pathway to regulate gene expression (Figure 4B).

### LKB1 functions with SMARCA4 to regulate lipid metabolism

**FIGURE 5:**
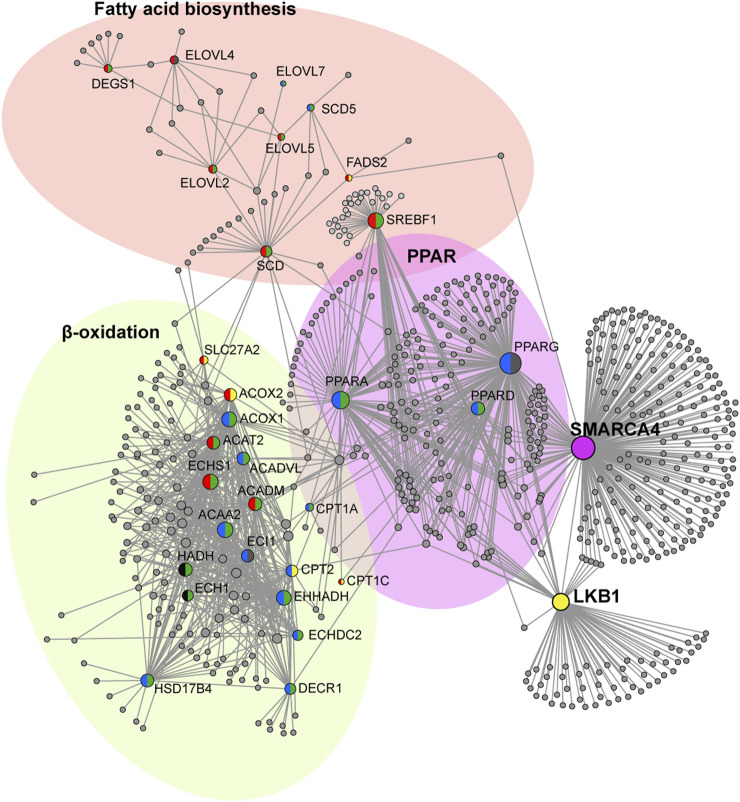
LKB1 and SMARCA4 collaborate to regulate lipid metabolism. Network maps of STRING interactions of genes involved in FA biosynthesis, PPAR, and β-oxidation. Nodes represent genes identified in GSEA. Node semi-circle colors represent up- or downregulation and DEG classification as follows: yellow = L-DEGs, purple = S-DEGs, green = LS-DEGs, red = upregulated, blue = downregulated, black = inconsistent expression profile between *L*vsWT, *S*vsWT, and *LS*vsWT, and gray = classification other than L-DEG, S-DEG, or LS-DEG. Whole yellow circles represent LKB1, whole purple circles represent SMARCA4, and whole gray circles represent protein interactors experimentally determined. Generic PPI with medium confidence stringency (600) and experimental evidence was used (Szklarczyk et al., 2019).

During a state of energy imbalance, when the AMP: ATP ratio is high, LKB1 activates AMPK, promoting increased lipid catabolism and reducing lipid anabolism. This phenotype is observed in *LKB1*-mutant tumors, where *LKB1* catalytic activity is lost; tumors exhibit increased lipid accumulation, which is associated with diminished AMPK activity (Bhatt et al., 2019). To determine the impact of SMARCA4 on lipid metabolism, we focused on genes that comprised the lipid metabolic process BP term from GSEA of *LKB1-SMARCA4* double mutant tumors and examined expression in the scRNA-seq dataset (Figure 2B). In particular, we investigated how SMARCA4 influences LKB1-mediated regulation of AMPK, which is crucial for controlling lipid metabolism (Hawley et al., 2003; Shaw et al., 2004; Lizcano et al., 2004); DEGs were then analyzed using ExpressAnalyst (Ewald et al., 2024) to further classify genes into anabolic (FA biosynthesis) and catabolic (FA β-oxidation and PPAR) processes (Supplementary Table S8). We then constructed protein interaction network maps using NetworkAnalyst and the STRING database (Zhou et al., 2019; Szklarczyk et al., 2019) to visualize the pathway interactions (Figure 5). Genes involved in FA biosynthesis were upregulated when *LKB1* or *SMARCA4* was mutated, consistent with previous observations that *LKB1* loss leads to the upregulation of FA biosynthesis (Faubert et al., 2014). We also observed that *SREBF1*, the gene coding for *SREBP1*, was upregulated and transcriptionally regulated by LKB1 and SMARCA4 (Supplementary Table S9). Conversely, genes involved in β-oxidation of FAs were downregulated, with LKB1 and SMARCA4 contributing to regulation of β-oxidation genes (Supplementary Table S8; Figure 5). We also observed that peroxisome proliferator-activated receptors (*PPARα *and* PPARγ*), nuclear receptors that bind to fatty acids and regulate gene expression involved in β-oxidation, were downregulated (Supplementary Table S8; Figure 5) (Lu et al., 2022). These results suggest that LKB1 and SMARCA4 cooperate to regulate FA biosynthesis and β-oxidation and that loss of *SMARCA4* exhibits a gene expression pattern correlated with *LKB1* loss.

## Discussion

Our results suggest that tumor suppressor functions of LKB1 and SMARCA4 are interconnected and that they may cooperate to regulate gene expression involved in diverse biological processes in lung cancer. We found that loss of either *LKB1* or *SMARCA4* results in similar transcriptional profiles, with genes involved in metabolism, chromatin organization, cell cycle, and immune response showing similar gene expression profiles. Finally, we validated our scRNA-seq results using human lung tumor bulk RNA-seq data from the cBioPortal tumor database, implicating the LKB1–SMARCA4 interaction in lung tumorigenesis.

**FIGURE 6:**
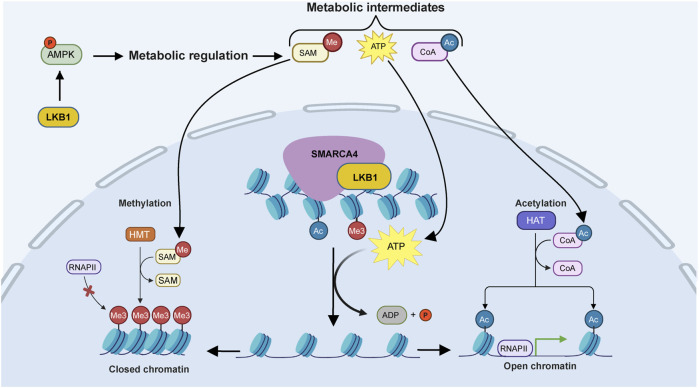
LKB1 and SMARCA4 cooperate to regulate chromatin remodeling and gene expression. Proposed model for LKB1 and SMARCA4 regulation of gene expression. LKB1 functions in the cytoplasm to regulate metabolic pathways through AMPK phosphorylation and activation. Metabolic intermediates ATP, acetyl-CoA, and SAM translocate to the nucleus where LKB1 stimulates the ATPase activity of SMARCA4 at target loci determined by histone modifications to disrupt nucleosomes and modify chromatin organization. The remodeled chromatin can then be further modified through epigenetic modifications using metabolic intermediates from LKB1-regulated pathways as cofactors. Histone methyltransferases (HMTs) can use SAM to promote histone methylation (ME3), creating closed chromatin and blocking transcription. Histone acetyltransferases (HATs) can use acetyl-CoA to acetylate histones (Ac), creating open chromatin and promoting transcription. Image created using BioRender and processed in adobe illustrator.

We propose a model for LKB1–SMARCA4-mediated transcriptional regulation based on our findings and suggest that LKB1 has two master regulatory functions: the first is as a master regulatory kinase regulating energy metabolism and the availability of cofactors used for chromatin remodeling, and the second is as a transcriptional regulator in complex with SMARCA4 in the nucleus to regulate chromatin remodeling and gene expression (Figure 6) (Mével-Aliset et al., 2025).

*LKB1*-mutant lung tumors exhibit increased lipid accumulation due to attenuation of AMPK signaling. In an ATP-deprived state (when the AMP: ATP ratio is high), LKB1 phosphorylates and activates AMPK, leading to inhibition of anabolic pathways and promotion of catabolic pathways (Bourouh and Marignani, 2022). When *LKB1* is mutated, genes involved in lipid biosynthesis are upregulated, and those involved in β-oxidation are downregulated (Figure 5). FA synthesis is regulated, in part, by AMPK phosphorylation of SREBP1, which is a transcription factor regulating the expression of genes involved in FA synthesis (Seo et al., 2015; Li et al., 2011). We observe that genes involved in FA biosynthesis are also upregulated when *SMARCA4* is lost, even in the presence of LKB1, suggesting that SMARCA4 is epistatic to LKB1 in regulating gene expression of FA biosynthesis genes (Figure 5). SMARCA4 has also been shown to play a role in the expression of genes involved in FA biosynthesis (Figure 5) (Li et al., 2018b). In hepatocytes, SREBP1 has been shown to bind the SMARCA4-dependent SWI/SNF complex, suggesting that FA biosynthesis gene expression is regulated by LKB1 and SMARCA4 within a linear pathway (Li et al., 2018b).

LKB1 has been shown to interact with SMARCA4 in the nucleus (Marignani et al., 2001), and loss of this interaction leads to global downregulation of gene expression (Mével-Aliset et al., 2025). Furthermore, our laboratory has shown that LKB1 binds to and promotes the ATPase-dependent chromatin remodeling function of SMARCA4 (Marignani et al., 2001). Therefore, we speculate that the striking correlated gene expression profiles that result when either *LKB1* or *SMARCA4* is mutated may be due to the absence of the LKB1–SMARCA4 physical interaction (Marignani et al., 2001). Individual functions of LKB1 and SMARCA4 in regulating gene expression are evident from L-DEGs and S-DEGs implicated in diverse pathways, but LS-DEGs consistently represent the predominant classification (Figure 4B; Supplementary Figure S3A). Our findings support the hypothesis that the primary mechanism by which LKB1 regulates gene expression is through the previously observed physical interaction with SMARCA4, whereby the LKB1–SMARCA4 interaction regulates the recruitment of the transcriptional and epigenetic regulators to gene regulatory elements mediated by binding with SMARCA4, promoting the ATPase activity and chromatin remodeling function of SMARCA4 (Marignani et al., 2001).

LKB1 metabolic regulation can also impact the chromatin landscape independently of SMARCA4 (Scott et al., 2007; Zeng and Berger, 2006; Bungard et al., 2010; Hou et al., 2011). Therefore, our observations may result from independent yet collaborative pathways that converge on epigenetic modification pathways, in addition to the previously observed LKB1–SMARCA4 interaction (Scott et al., 2007; Zeng and Berger, 2006; Bungard et al., 2010; Hou et al., 2011). Metabolic pathways produce intermediates such as ATP, acetyl-CoA, and SAM, which are used as cofactors for chromatin remodeling enzymes that regulate global changes in chromatin organization and consequently gene expression (Gut and Verdin, 2013; Kottakis et al., 2016; Lee et al., 2014). The metabolic state of cells influences the abundance of these cofactors. For example, lipid metabolism drastically impacts the levels of ATP and acetyl-CoA through regulation of FA biosynthesis and β-oxidation (Sutendra et al., 2014). Conversely, SAM production is dependent on the serine–glycine one-carbon cycle (Kottakis et al., 2016). *LKB1* loss is associated with global chromatin remodeling and increased histone methylation through upregulation of SAM production (Kottakis et al., 2016; Pierce et al., 2021). Metabolic intermediates from LKB1-regulated pathways may be utilized by chromatin remodeling complexes to regulate epigenetic modifications. Moreover, chromatin remodeling complexes can interact with epigenetic enzymes, regulating histone modifications, and in this way, LKB1 can regulate epigenetic modifications indirectly from SMARCA4 (Nakatsuka et al., 2017; Januario et al., 2017). We suggest that LKB1 functions as a master regulator of gene expression through regulation of metabolic pathways, production of epigenetic cofactors, and modulation of chromatin remodeling via its physical interaction with SMARCA4. Since LKB1 can traverse the nuclear membrane (Nezu et al., 1999), where it can then be recruited to transcriptional machinery through direct binding within the nucleus, LKB1 could function as an integrator of the metabolic state of cells to responses in gene expression, impacting the production of ATP and other metabolic intermediates utilized by SMARCA4 and the chromatin remodeling machinery to regulate chromatin structure and, consequently, gene expression (Figure 6).

In summary, we show that *LKB1* and *SMARCA4* loss exhibit similar expression profiles in lung cancer cell lines and human lung tumors and that this interaction contributes to the regulation of diverse pathways in lung cancer. We propose a model in which the tumor suppressors LKB1 and SMARCA4 cooperate to prevent lung cancer through both direct and indirect mechanisms that converge to regulate metabolism and gene expression. Our results provide evidence that implicates LKB1 as a master regulator, serving as an interface between metabolic pathways and gene expression. We propose that this function of LKB1 is mediated, in part, through its physical interaction with SMARCA4, positioning LKB1 as a nexus between metabolism and gene expression.

## Methods

### Workflow to identify LKB1 (L), SMARCA4 (S), and LKB1–SMARCA4 (LS) DEGs

scRNA-seq data were mined and processed as in our previous study (Kim et al., 2021). All DEGs detected from scRNA-seq were identified, and the log_2_FC expression level for each gene was determined with Calu-3 cells serving as a control: H460 vs. Calu-3 (*L*), H1299 vs. Calu-3 (*S*), and A549 vs. Calu-3 (*LS*). Classification of DEGs was as follows: L-DEGs were detected in *L* and *LS,* not in *S*. S-DEGs were detected in *S* and *LS,* not in *L*. LS-DEGs were detected in *L*, *S*, and *LS*. Each classification represented a gene set for gene set enrichment analysis.

### Data processing and gene set enrichment analysis

For gene set enrichment analysis, we used g:Profiler (Kolberg et al., 2023) to identify enriched GO terms at the category of BP using default settings. Results from upregulated, downregulated, and complete gene sets were combined, and redundant BP terms were filtered for the highest intersection size. Enriched BP terms were then compared with each other using a similarity matrix based on overlapping gene IDs. Hierarchical clustering was performed using enriched BP terms via hierarchical k-means clustering (Silhouette method) to identify unique GeneIDs for each cluster. The similarity matrices and hierarchical clustering were generated using RStudio. Unique gene IDs for each cluster were then analyzed using the ExpressAnalyst (Liu et al., 2023) network enrichment tool with the Panther database to generate enriched BP terms for dot plots, with only terms with >3 gene IDs (hits) chosen and only statistically significant term results collected (FDR-corrected *p*-value<0.05). Genes that are annotated to lipid metabolic process, chromatin organization, cell cycle, and immune response GO terms were obtained from the Gene Ontology Resource (Ashburner et al., 2000; Aleksander et al., 2023).

### Plots

Venn diagrams were generated using the Ghent University Venn diagram generator web tool. Heatmap plots were generated using the Broad Institute Morpheus web tool. Volcano and dot plots were generated using ggplot2 in RStudio. Network maps were generated using NetworkAnalyst (Zhou et al., 2019) and processed in Adobe Illustrator.

### Correlation analysis using scRNA-seq and bulk RNA-seq lung cancer datasets

We obtained a transcripts per million (TPM)-normalized mRNA expression matrix of 517 bulk RNA-seq datasets from patients with lung adenocarcinoma (LUAD) identified in The Cancer Genome Atlas (TCGA) and Broad Genome Data Analysis Center (GDAC) Firehose in the cBioPortal for the Cancer Genomics database (Cerami et al., 2012). For the correlation analysis, we selected 66 datasets based on the mutation status of three tumor suppressor genes, namely, *LKB1*, *SMARCA4*, and *TP53*, and divided them into four subgroups that are represented by genotypes similar to the cell lines used for scRNA-seq: Calu-3 represented by *TP53*-mutant tumors (Zhou et al., 2019), H460 represented by *LKB1*-mutant tumors (Gao et al., 2022), H1299 represented by *SMARCA4*-mutant tumors (Kottakis et al., 2016), and A549 represented by *LKB1*-*SMARCA4* double mutant tumors (Gut and Verdin, 2013). A TPM-normalized mRNA expression matrix of the 66 subgroup datasets was used for hierarchical clustering analysis following a principal component analysis for dimension reduction. Finally, gene expression-based relationships among the four lung cancer patient subgroups (bulk RNA-seq) and the four lung cancer cell lines (scRNA-seq) were respectively identified using the Pearson-correlation method. In addition, we prepared two separate TPM-normalized mRNA expression matrices from bulk RNA-seq datasets of LUAD patients for 299 early-stage samples (237 WT, 7 *LKB1*-mutated, 3 *SMARCA4*-mutated, and 51 *TP53*-mutated) and 83 late-stage samples (61 WT, 2 *LKB1*-mutated, 2 *SMARCA4*-mutated, and 18 *TP53*-mutated). A DEA was performed on each of the two mRNA expression matrices to detect marker genes significantly up- or downregulated in mutated samples of each tumor suppressor gene at the two different lung cancer stages compared to WT samples. To select significant marker genes, we applied identical parameters that were used for the DEA of scRNA-seq datasets.

## Data Availability

The original contributions presented in the study are included in the article/Supplementary Material; further inquiries can be directed to the corresponding author.
