# Supplementary figures and images for: Tumor suppressors LKB1 and SMARCA4 functionally interact to regulate gene expression across diverse biological processes in lung cancer
Source: Front Cell Dev Biol. 2026 Mar 17;14:1685342. doi: 10.3389/fcell.2026.1685342 (PMC13036190; doi:10.3389/fcell.2026.1685342)

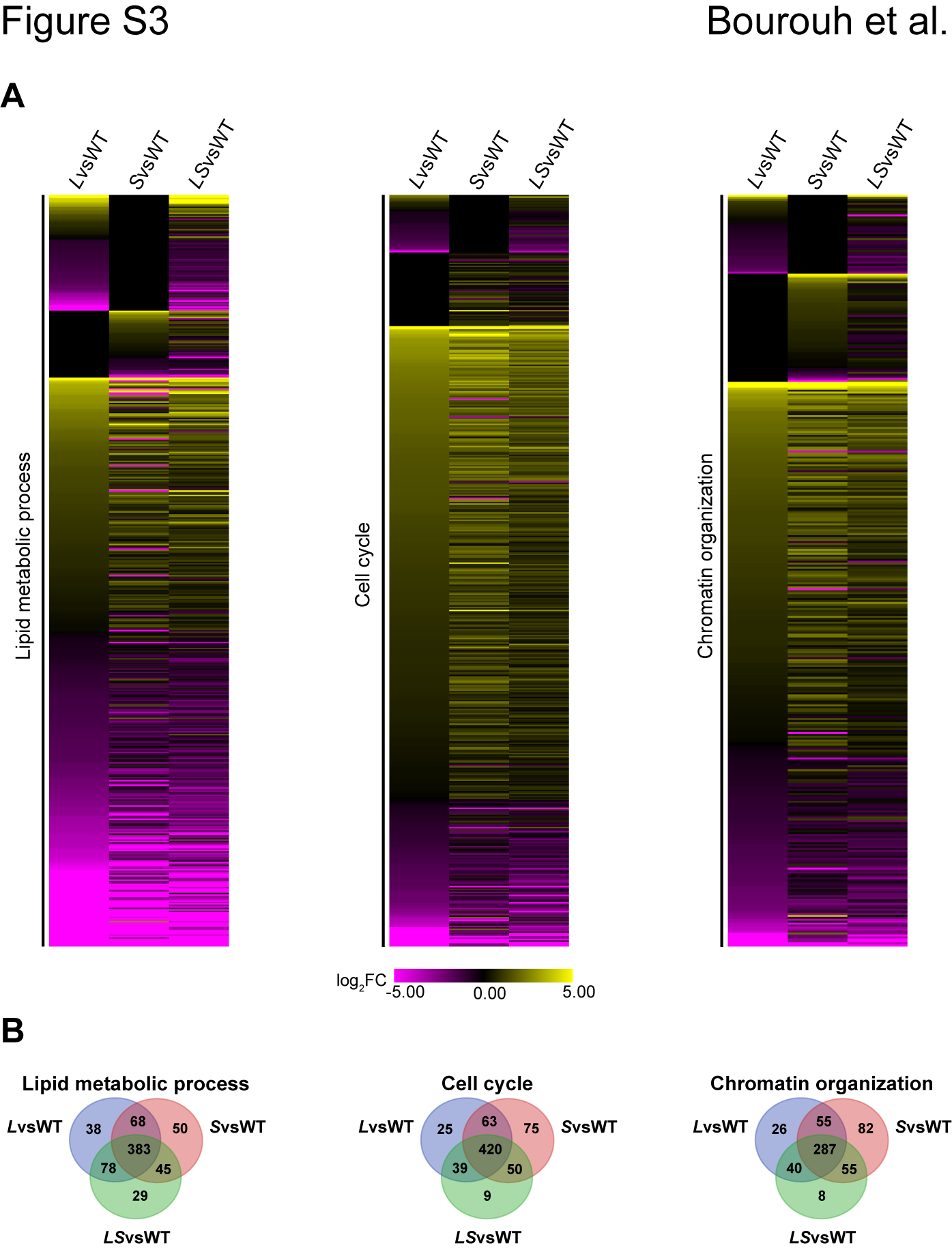

Supplement: Supplementary file 4 [file Image3.tif]

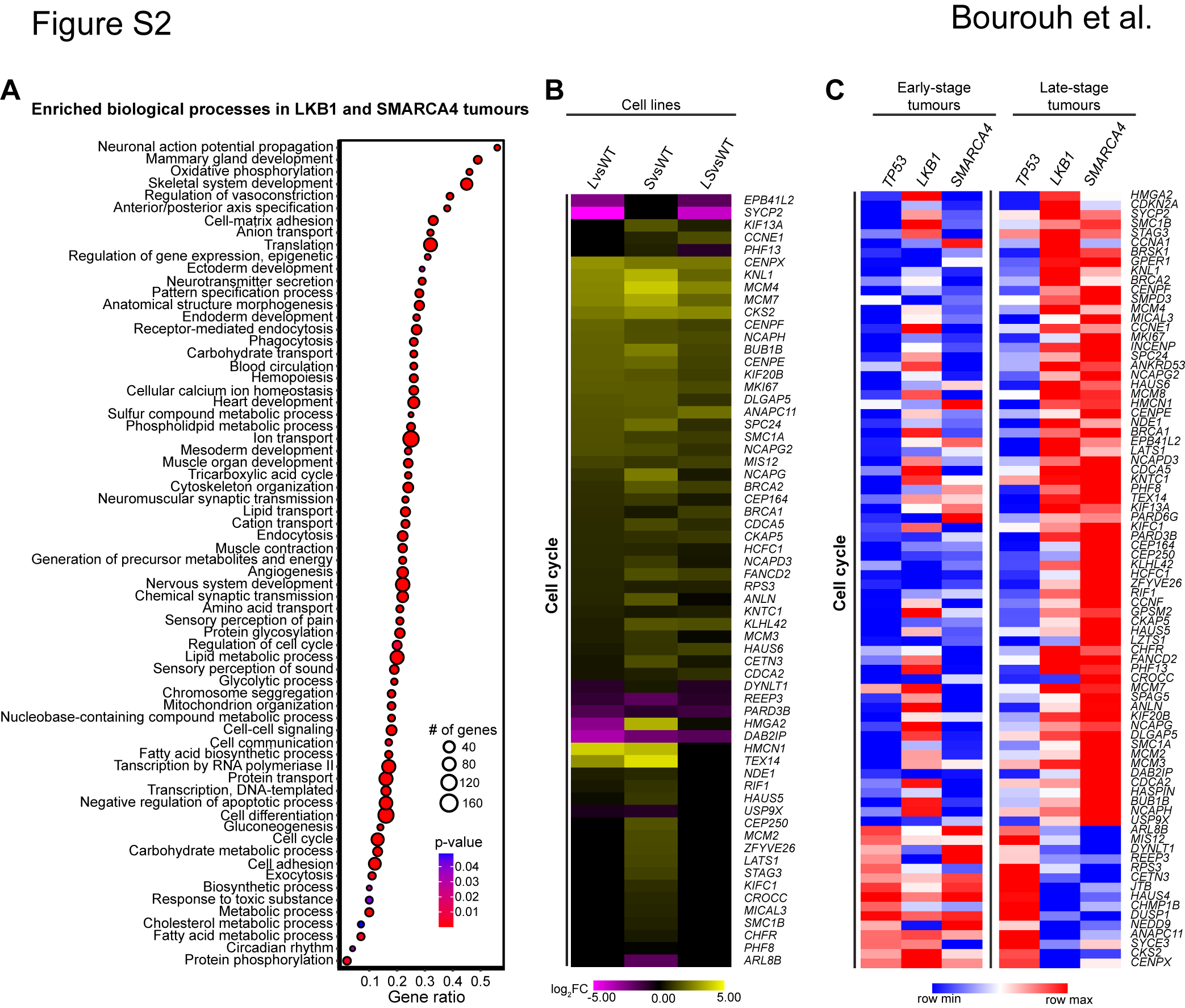

Supplement: Supplementary file 5 [file Image2.tif]

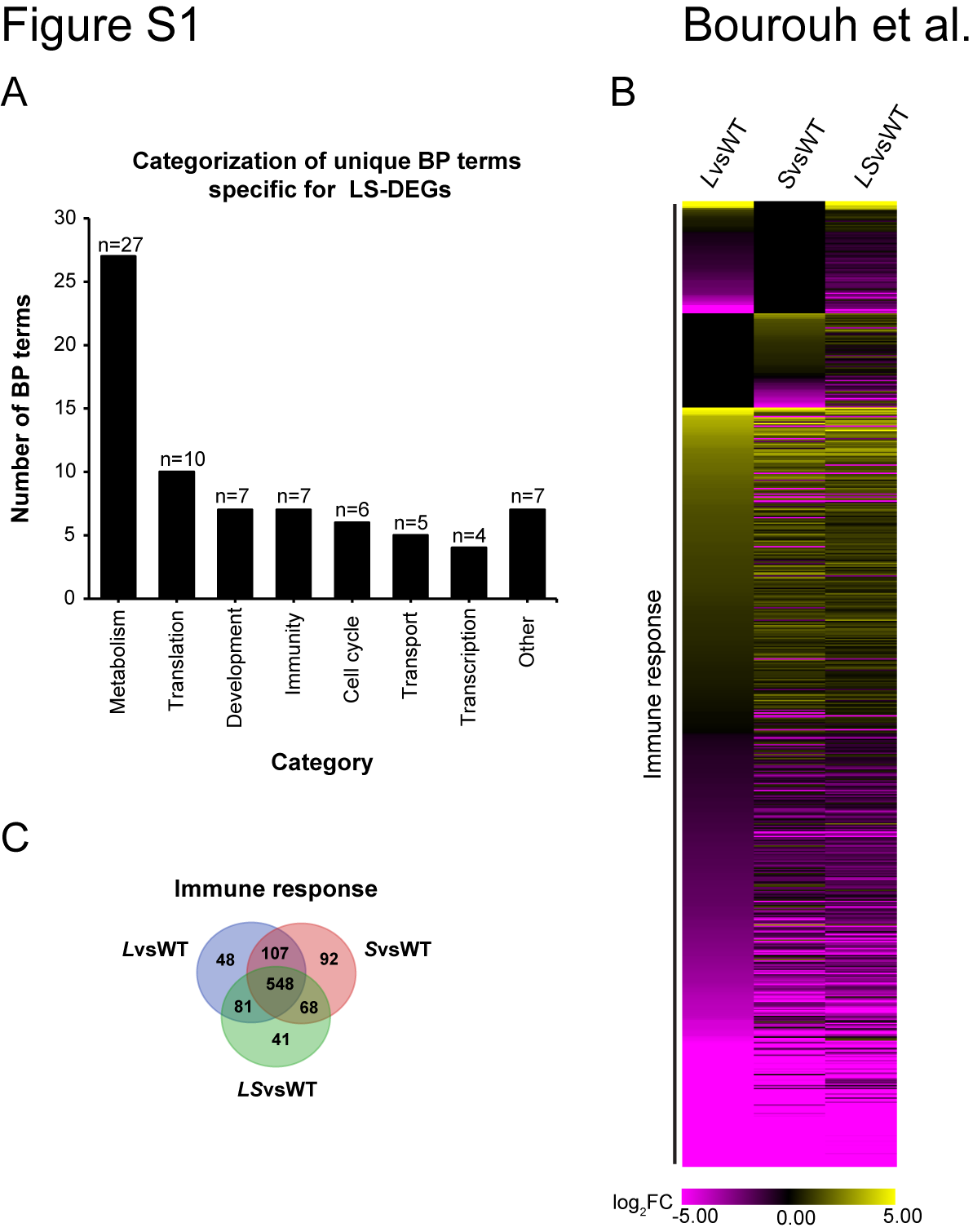

Supplement: Supplementary file 6 [file Image1.tif]
